# 26.1 MOTOR SUBTYPES AND PREDICTION OF COURSE IN PSYCHOSIS RISK YOUTH

**DOI:** 10.1093/schbul/sby014.106

**Published:** 2018-04-01

**Authors:** Vijay Mittal, Derek Dean, Sebastian Walther, Tina Gupta, Teresa Vargas, Juston Osborn

**Affiliations:** 1 Northwestern University; 2 University of Colorado Boulder; 3 University Hospital of Psychiatry

## Abstract

**Background:**

Prominent etiological conceptions of psychosis implicate abnormal cortico-striatal circuits. Dysfunction in these critical systems, responsible for filtering information and modulating higher-order function, may account for heterogeneous presentations of symptoms and characteristics of psychosis. Collectively, a body of work from our group and from other teams indicates that evaluating select motor behaviors and abnormalities, which directly reflect function of these circuits, may be a useful method for understanding and predicting the neural underpinnings of psychosis. In the context of the psychosis risk period, partitioning clinical high-risk (CHR) youth based on objective behavior may help guide early detection and intervention efforts, and provide a novel perspective on different etiological pathways or patient subtypes.

**Methods:**

Using an unsupervised machine learning approach, 69 CHR young adults were included in a K-means cluster analysis based on their performance on instrumental measures of psychomotor slowing, dyskinesia, and neurological soft signs (NSS)—distinct motor domains affected across the psychosis spectrum. We also recruited a group of 70 matched healthy controls (HC) for comparison. All participants were also assessed with a resting-state functional connectivity analysis (rcfMRI). The resulting CHR group clusters and HCs were then compared on positive and negative symptoms, multiple cognitive domains, and cortical-striatal seed based resting state analysis.

**Results:**

Results of a 3-cluster solution suggest that there are subtypes of CHR individuals who show psychomotor slowing, average motor performance, and impairment on measures of dyskinesia as well as NSS domains for motor coordination, sequencing and sensory integration. The cluster of individuals showing dyskinesia and abnormal NSS also have more severe negative symptoms and impairment on a number of cognitive domains. Furthermore, the clusters of CHR individuals who show psychomotor slowing and the cluster showing dyskinesia and abnormal NSS have different cortical-striatal connectivity compared to UHR who show average motor behavior and healthy controls.

**Discussion:**

These results provide evidence for etiological theories highlighting altered cortico-striatal networks and the importance of examining motor behavior prior to the onset of psychosis. Taken together, this approach may reflect a novel strategy for promoting tailored risk assessment as well as future research developing individualized medicine.

